# ARTEMIS: An Explainable AI Framework for Multi-Class COVID-19 Diagnosis with a Newly Curated Dataset

**DOI:** 10.3390/bioengineering13050588

**Published:** 2026-05-20

**Authors:** Muhammet Emin Sahin, Hasan Ulutas, Mustafa Fatih Erkoc, Baris Karakaya, Recep Batuhan Günay, Enes Eren Suzgen

**Affiliations:** 1 Department of Computer Engineering, Izmir Bakırçay University, Izmir 35665, Türkiye; 2 Queen Mary’s Digital Environment Research Institute (DERI), London E1 1HH, UK; 3 Department of Computer Engineering, Yozgat Bozok University, Yozgat 66100, Türkiye; 4 Department of Radiology, Faculty of Medicine, Yozgat Bozok University, Yozgat 66100, Türkiye; 5 Department of Electrical Electronics Engineering, Faculty of Engineering, Firat University, Elazig 23119, Türkiye; 6 Department of Computer Technologies, Sorgun Vocational High School, Yozgat Bozok University, Yozgat 66700, Türkiye

**Keywords:** COVID-19, deep learning, Explainable AI (Grad-CAM++), CT, X-ray

## Abstract

In this work, we propose ARTEMIS, a novel and highly interpretable deep learning pipeline for the automatic classification of Chest X-ray (CXR) and Computed Tomography (CT) images into different categories related to important clinical outcomes: COVID-19 infection, Community-Acquired Pneumonia (CAP) cases, and Normal cases. Unlike existing models based on the static feature enhancement step, ARTEMIS proposes a learnable preprocessing component that dynamically adapts the image contrast and sharpness in training mode, facilitating adaptive optimization. Our hybrid network combines EfficientNet-B0 backbone with built-in SE attention with the optional lightweight Transformer encoder block to jointly learn local radiological features and global relationships between pixels. Comprehensive experiments have been conducted on five different datasets, which comprise four publicly available ones and one novel CT dataset annotated by radiologists, including X-ray and CT modalities. Experimental results show strong robustness and generalization with macro F1-scores greater than 96% on public datasets and 99.39% accuracy on our new CT dataset. To interpret the decision-making process, Grad-CAM++ is employed to generate class-discriminative saliency maps; the highlighted regions are systematically validated against established radiological criteria by a board-certified radiologist, confirming that model decisions are grounded in clinically meaningful pulmonary findings rather than imaging artifacts.

## 1. Introduction

COVID-19, often termed coronavirus disease 2019 due to being caused by the severe acute respiratory syndrome coronavirus 2 (SARS-CoV-2), can be considered among the most tragic epidemics in recent history, having impacted millions of people throughout the world since its discovery. By early March 2020, this epidemic became declared a pandemic on a global level [1,2]. The number of infected patients increased drastically, and the mortality rate followed the same trend, with global figures reflecting millions of infections, considerable fatalities, and a large share of recovered cases worldwide [3]. The negative socioeconomic impacts of this epidemic, including a part played in the creation of global financial instabilities, still resonate across the globe. In epidemics of such nature, the resiliency of the national healthcare system and advanced technologies for diagnosis become critically important [4]. After the appearance of the novel coronavirus, a vast number of chest X-ray datasets started appearing in the literature to shed light on the impact of the virus on the lungs and help physicians in diagnosis. In this regard, the use of artificial intelligence (AI) and deep learning algorithms becomes particularly prominent due to advances in big data and technological innovation.

It is notable that machine learning and deep learning algorithms have found widespread application in various fields through processing large datasets, including disease diagnosis and image classification [5,6,7,8,9,10], erosion prevention in composite insulations [11], criminal activity prediction [12], and automatic answer selection in computer networks [13]. Deep learning algorithms used in such studies continuously evolve towards increasing performance in classification and recognition tasks. A large body of scientific works have addressed the problem of detecting COVID-19 from chest X-ray images using pretrained deep learning models. Specifically, Kong et al. [14] proposed a method involving combination of features of both DenseNet and VGG16 along with image segmentation using ResNet, resulting in a record-breaking 98% classification accuracy. Nayak et al. [15] presented a fast and accurate system aimed at providing an alternative to PCR tests for detecting COVID-19 through the comparison of a lot of different deep learning models (Inception-V3, AlexNet, ResNet-34, VGG-16, GoogleNet, MobileNet-V2, ResNet-50, and SqueezeNet); the best results were attained by ResNet-34, with a classification accuracy of 98.33%. Chowdhury et al. [16] analyzed the capability of AI to provide for rapid and highly accurate diagnoses for COVID-19 through processing chest X-ray images; classification success rate equaled 99.7% in their study, in which they used a database with 423 images with COVID-19, 1485 with pneumonia, and 1579 normal images. Narin et al. [17] tested five different deep learning algorithms on three databases within a four-class classification framework (COVID-19/Healthy/Viral Pneumonia/Bacterial Pneumonia), achieving the best results with ResNet50. Despite the efficiency of well-trained deep learning algorithms, the process of input image preprocessing plays a vital role in the success, because the better prepared the input data are, the better the results tend to appear. As a result, novel approaches for data preprocessing are continuously introduced. For example, Wei et al. [18] developed a data preprocessing approach to increase the performance of the collected dataset in terms of device-free localization using deep learning, achieving 18% improvement in RMSE of localization. In another study, researchers provided a review of medical ultrasound (US) image data preprocessing techniques, including data augmentation, noise reduction, and image enhancement; according to the authors, the appropriate preprocessing helps achieve better results even with relatively small-size datasets [19]. Asıf et al. [20] designed a robust transfer learning approach for brain tumor detection on the basis of MRI, incorporating data preprocessing in the form of data augmentation and the usage of three optimizers (ADAM, SGD, and RMSprop), along with L2 regularization; the best results appeared with a CNN model based on the Xception architecture with the ADAM optimizer. Speaking about COVID-19 detection, Basu et al. [21] analyzed whether additional components in the preprocessing step can boost performance of the deep image classifiers through examining seven traditional classifiers and the influence of preprocessing on them; in particular, they explored the impact of preprocessing on transfer-learned classifiers for respiratory disease diagnosis using lung radiographs. More recently, Yaman S. et al. [22] implemented a new MVSR normalization algorithm for chest X-rays to increase the performance of the CNN classifier; with this technique implemented in the embedded platforms, the classifier achieved 96.16% classification accuracy against 83.01% before the preprocessing. The use of deep learning algorithms proved to be quite effective in automating the process of diagnosing medical images during recent years in the context of the COVID-19 pandemic; however, most studies were limited to using only one kind of data and fixed preprocessing pipelines. Moreover, publicly available datasets were biased, unbalanced, or had low annotation quality. The present study addresses three interconnected gaps that remain unresolved in the existing literature. First, the vast majority of published COVID-19 AI models are validated on a single dataset and a single imaging modality, raising legitimate questions about cross-dataset generalizability. Second, existing preprocessing pipelines are static—they apply identical transformations to every image regardless of its quality, contrast, or acquisition protocol, discarding potentially useful diagnostic information in the process. Third, while explainability is frequently cited as a requirement for clinical AI, most studies either omit it entirely or provide heatmaps without systematic radiological validation. ARTEMIS is designed to address all three limitations simultaneously: a single unified framework is evaluated across five heterogeneous datasets spanning two modalities (CT and X-ray), a learnable preprocessing module adapts to each image’s individual distribution rather than applying fixed transformations, and Grad-CAM++ visualizations are validated against established radiological criteria by a board-certified radiologist. Taken together, these contributions represent a substantive advance over existing single-dataset, static-preprocessing approaches, even where absolute accuracy figures may be comparable to the state of the art on individual benchmarks. Specifically, the contributions made can be stated as follows:

1. This study involves a comparison between the performance of one framework for diagnostic purposes across five heterogeneous datasets, including four public collections and one novel CT dataset developed specifically for the experiment.

2. The proposed technique maintains high diagnostic performance with accuracy and macro F1 scores above 96% in the case of using public datasets and up to 99.39% accuracy when working with the custom CT dataset.

3. The new, domain-specific dataset for diagnostics of COVID-19 patients was designed in the course of the study and annotated by experts; it contains classes of COVID-19, community-acquired pneumonia (CAP), and normal patients, and all of the per-class precision and recall are above 0.99.

4. A special experiment procedure was designed to compare results for both balanced and imbalanced datasets of different sizes, imaging modality (X-ray vs CT), and class composition; all of the standard deviations of accuracy in these experiments were less than 1.2%.

5. Model performance was measured in a number of quantitative metrics, including accuracy, precision, recall, class-wise F1 scores, macro averages, AUC curve areas, and confusion matrix. AUC values averaged near 0.99.

6. Model interpretability is provided through the implementation of Grad-CAM++ visualization of deep layer activation maps, which correspond to bilaterally located ground-glass opacities in COVID-19, localized consolidation in CAP, and relatively normal parenchyma in Normal cases.

The rest of the paper is structured as follows. Section 2 describes materials and methods, focusing particularly on the custom dataset development, data preparation and preprocessing techniques, and the ARTEMIS framework architecture. The experimental procedure, hyperparameters tuning, and evaluation metrics are outlined in Section 3, along with the results obtained using this framework on different datasets with visual explanations based on Grad-CAM++. Discussion and conclusions are provided in Section 4 and Section 5, respectively.

## 2. Material and Methods

### 2.1. Dataset

**Figure 1:**
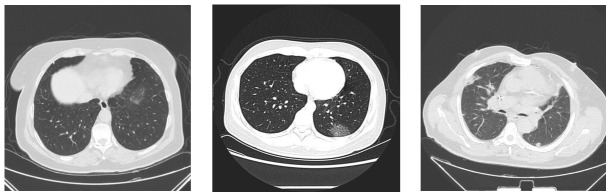
Samples from Dataset.

The proposed study used a new dataset consisting of chest CT scans obtained from Bozok University Medical Faculty Hospital (Yozgat, Türkiye). The collection of data took place between March 2020 and October 2021, covering both waves of the COVID-19 epidemic [23]. In total, 18,000 axial CT slices were used. These slices were retrospectively retrieved, anonymized, and split into the following classes: COVID-19 pneumonia (n = 6000), community-acquired pneumonia (CAP, n = 6000), and normal lungs (n = 6000). In the dataset from [24], we used only CAP examples, which resulted in creating a new dataset with multiple classes. Each scan was annotated by two board-certified radiologists; see Figure 1 below. Any inconsistencies in labeling were discussed, and an agreement was achieved. The confirmation of the diagnosis of COVID-19 was carried out using RT-PCR tests and the patient’s clinical history. The selection of CAP cases was based on imaging findings, clinical features, and the results of other microbiological examinations (excluding COVID-19). Normal lungs cases were taken from routine scans of healthy individuals whose clinical and radiological examination excluded pulmonary diseases. All of the images were kept in the DICOM format and then transformed into PNG images for further use in machine learning models after normalization. The original image size was approximately 512 × 512 pixels with the voxel spacing equal to 1 mm if such slices were available. Multiple CT scans from the same patient were assigned the same label, and patients were divided into training, validation, and test groups.

Apart from the dataset that was generated in the current research, multiple freely available datasets, which are commonly used in the literature, were considered; these datasets include CT scans as well as chest radiography images. The Large COVID-19 CT Slice Dataset, the COVID-19 Radiography Database, as well as two chest X-ray datasets obtained on Kaggle (Amanullah Asraf and Sachin Kumar) [25,26,27,28] were used. The characteristics of these datasets and their role in the experimentation will be discussed in the following section. First, all datasets were divided in 80%/20% ratio for training and testing purposes. Then, the datasets for training were subdivided in 75%/25% ratio for training and validation, respectively.

### 2.2. Data Preprocessing

Unlike classic approaches relying on fixed pre-processing steps (e.g., contrast normalization or histogram equalization), ARTEMIS utilizes a trainable image enhancement block that adapts the contrast and sharpness of the input image during training. The ability to perform such operations helps the algorithm adaptively highlight diagnostic-relevant areas, like ground-glass opacities or lobar consolidations, making it easier to classify the corresponding features. As a result, besides classifying the input image, ARTEMIS provides a tailored-to-task image pre-processing pipeline. In standard medical imaging workflows, preprocessing steps (e.g., histogram equalization, Gaussian blurring, or window-level tuning) are performed statically and independently from the training process. This work proposes an alternative method for image enhancement using a trainable pre-processing pipeline. Specifically, the preprocessing layer is made up of two differentiable components:

**Contrast Enhancement:** Using a learnable scalar value, the contrast between the pixels of the input image and its mean intensity is adjusted.

**Unsharp Mask with Depthwise Gaussian Filter:** Subtracting the output of the depthwise Gaussian filter from the contrast-boosted input yields high-frequency features.

The two steps described above are combined into a single convolutional layer called PreprocessingLayer. This layer is trained simultaneously with the entire model to enhance the input image based on the task at hand. Although the learnable preprocessing, SE attention, and Transformer components are architecturally general, their configuration in ARTEMIS is specifically motivated by the characteristics of pulmonary CT and X-ray imaging. In chest CT, ground-glass opacities and consolidations appear as subtle, low-contrast density variations easily overlooked by fixed preprocessing pipelines. The DCAP module’s contrast and sharpness parameters are optimized end-to-end to amplify these diagnostic cues. HU windowing (hu_min_ = −600, hu_max_ = 150) constitutes a lung-specific normalization restricting the dynamic range to the pulmonary window. SE attention within the EfficientNet-B0 backbone recalibrates channel responses, systematically suppressing artifact-related channels while boosting parenchymal texture encoding. The Transformer captures long-range spatial dependencies across bilateral lung fields—critical for COVID-19, where bilateral involvement is a defining radiological criterion.

### 2.3. Deep Learning

Deep learning may be considered a subspecialty within machine learning that involves training models capable of automatically extracting hierarchical features from raw input through stacked architectures, such as artificial neural networks. In contrast to conventional techniques involving extensive feature engineering, deep learning methods and especially CNNs have proven to achieve high levels of success in performing difficult tasks, including image classification, speech recognition, and even medical diagnosis [29,30]. Deep learning models have increasingly found use in the domain of medical imaging due to their superior feature representation capability and higher precision when compared with traditional computer vision algorithms [31]. To further strengthen feature robustness, the ARTEMIS backbone adopts principles from robust representation learning [32], ensuring that features remain discriminative across heterogeneous imaging conditions. Additionally, the model’s reliability under noisy or adversarial input conditions is reinforced through design choices informed by proactive detection frameworks [33].

The current paper introduces an innovative deep learning architecture composed of a trainable preprocessing step, EfficientNet-B0 backbone, and optional light-weight Transformer block aimed at achieving improved classification results and interpretability of models for medical imagery analysis. The learnable preprocessing component utilizes differentiable contrast enhancement and sharpening functions, thus allowing for data-driven tuning of early-stage visual features. Next, an EfficientNet-B0 backbone pretrained on ImageNet-1K is used as the primary feature extractor. EfficientNet-B0 employs MBConv blocks with built-in squeeze-and-excitation (SE) attention, providing efficient and discriminative feature extraction with minimal computational overhead. Finally, if needed, a specialized attention mechanism can be employed to consider long-range dependencies in the image. Together with the aid of explainability algorithms such as Grad-CAM++ and data augmentation, the architecture described here provides for highly accurate and transparent diagnoses.

### 2.4. Hyperparameter Optimization

Hyperparameter optimization refers to the practice of finding the best configuration of hyperparameters for a learning algorithm in order to optimize its performance in a specific task. Unlike the parameters of a model obtained through training, hyperparameters are predefined before the training procedure and significantly affect the convergence rate and generalization ability. Hyperparameter tuning is nowadays considered an important element in deep learning pipelines [34,35]. In the present study, the hyperparameter optimization process was carried out utilizing the Optuna v4.2.1 package, a modern optimization library developed to assist in hyperparameter tuning through efficient approaches such as Tree-structured Parzen Estimators (TPE) [36]. Hyperparameters, mainly the learning rate, weight decay, dropout rate, and architecture design features like whether or not to use transformers, were optimized with the goal of improving the proposed approach’s performance and robustness.

### 2.5. Evaluation Metrics

In this study, we evaluate the efficiency of the proposed classification model based on several classification measures that are commonly used for analyzing medical images. These measures comprise accuracy, precision, sensitivity (recall), F1 measure, and AUC. Due to the prevalence of class imbalance among clinical data, we also calculate macro-averaged and weighted F1 scores to provide an unbiased evaluation of the classifier across all classes. Furthermore, confusion matrix are utilized to visually represent the results predicted by the model against the actual values.

## 3. Experimental Results

This section discusses the results obtained during the implementation of the proposed model, which is a multiclass classification problem based on an artificial medical imaging dataset. These experiments serve to confirm the effectiveness of implementing the adaptive pre-processing module, the implementation of EfficientNet-B0 backbone and transformer architectures, and hyperparameters’ impact. For the sake of robustness and generalization, the stratified split of the dataset, augmentation of the training and validation sets, and testing of the models with and without TTA were performed. The computations were run on a workstation that had an NVIDIA RTX 4090 graphics card (22.5 GB memory) (NVIDIA Corporation, Santa Clara, CA, USA). Windows 11 Pro was the chosen operating system. The proposed architecture was coded using Python 3.11.11 and PyTorch 2.6.0 +cu118 frameworks. Other required software libraries include scikit-learn 1.6.1, OpenCV 4.11.0, Matplotlib 3.10.1, and Optuna v4.2.1.

### 3.1. Model Architecture and Technical Contributions

The proposed ARTEMIS model introduces a novel hybrid architecture that integrates convolutional and transformer-based components, with a strong emphasis on interpretability, adaptability, and generalization in medical image classification tasks.

#### 3.1.1. Architecture

**Figure 2:**
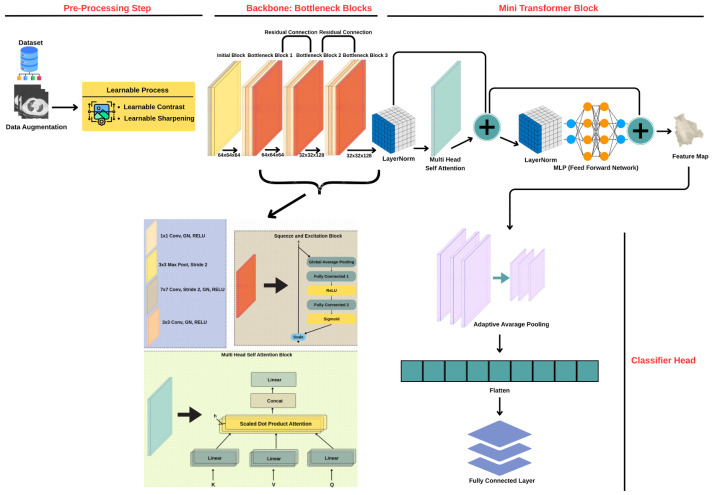
The overview of the model structure.

As shown in Figure 2 below, ARTEMIS begins with a preprocessing layer that is learnable for improving the contrast and sharpness and then followed by an EfficientNet-B0 backbone with built-in SE attention and an optional transformer encoder layer. In addition, the final classification layer uses the features obtained after global pooling for classification. Using such an approach allows the model to leverage not only the local texture cues but also global contextual cues from CT images.

#### 3.1.2. Learnable Preprocessing Layer

Unlike traditional, fixed preprocessing techniques, ARTEMIS incorporates a differentiable, trainable image enhancement layer. This module performs contrast amplification and unsharp masking via Gaussian-based filters or shallow convolutional blocks. Optimized end-to-end with the model, it enables ARTEMIS to learn optimal enhancement parameters that emphasize diagnostically relevant features (e.g., subtle opacities or edges), improving downstream feature extraction and classification.

#### 3.1.3. EfficientNet-B0 Backbone with SE Attention

The ARTEMIS feature extraction backbone is EfficientNet-B0 pretrained on ImageNet-1K. EfficientNet-B0 employs MBConv blocks with built-in squeeze-and-excitation (SE) attention, which adaptively recalibrates channel responses by boosting diagnostically relevant features (e.g., parenchymal texture encoding ground–glass opacities) and suppressing artifact-related channels (e.g., scanner noise, motion blur). This channel-wise recalibration incurs minimal computational overhead while substantially improving feature discriminability for pulmonary pathology.

#### 3.1.4. Adaptive Transformer Module

The ARTEMIS framework optionally includes a transformer-based encoder block after the convolutional feature extraction network. The encoder makes use of multi-head self-attention along with feed-forward blocks to learn long-term spatial relationships and global context in the image. The architecture of the transformer is residual, allowing its output to be concatenated with the convolutional features. A category of such hybrid models combining the properties of convolutional neural networks, which are sensitive to local features, and transformers, which reason about global relationships, has shown better performance in medical image analysis tasks. The inclusion of the Transformer block in ARTEMIS can be enabled/disabled using a hyperparameter.

#### 3.1.5. Training Strategies: MixUp and Regularization

Generalization and robustness are encouraged in ARTEMIS through the following sophisticated training approaches:

-MixUp data augmentation: convex linear combinations of inputs and their respective labels are utilized to facilitate smooth decision boundaries and prevent overfitting.

-Label smoothing: small probabilities are assigned to classes other than the ground truth to prevent overconfidence.

-Focal loss: during training, more weight is placed on difficult-to-classify samples, proving particularly effective in imbalanced datasets. These approaches minimize the memorization of noisy information and promote calibration, which becomes particularly relevant when dealing with medical images, where diversity is inherently constrained.

#### 3.1.6. Optimization: Cosine Annealing and Stochastic Weight Averaging (SWA)

Model training uses a learning rate schedule based on cosine annealing that gradually reduces the learning rate with the help of a cosine function, helping the model converge to a minimizer that is less sharp. SWA is then used during the final epochs for model training, where model weights are averaged over several checkpoints. This method provides a more smoothed loss surface with improved generalization performance without any additional computational cost. Both these methods have proven effective in deep learning model training, especially on noisy datasets.

#### 3.1.7. Explainability with Grad-CAM++

In order to increase transparency in the decision-making process, the ARTEMIS system uses Grad-CAM++, which is a more sophisticated technique that highlights the discriminative areas of the input image. As compared to the conventional Grad-CAM approach, Grad-CAM++ produces better-quality heat maps, making it highly desirable in complicated clinical scenarios where anomalies could be present in different areas of the lungs. In the context of this work, explainability refers to the ability of the model to identify and visualize the specific spatial regions of the input image that most strongly influenced its classification decision and to demonstrate that these regions correspond to clinically meaningful radiological findings rather than imaging artifacts or background structures. ARTEMIS achieves this through Grad-CAM++, which computes class-discriminative localization maps by computing the gradient of the predicted class score with respect to the final convolutional feature map. The resulting heatmaps are overlaid on the original image to produce spatial attention visualizations. Crucially, the explainability claim in this paper is not limited to producing heatmaps—it is substantiated through the systematic comparison of the highlighted regions against established radiological criteria: bilateral subpleural GGOs for COVID-19, lobar consolidation for CAP, and parenchymal clarity for Normal cases (see Section 4). This radiologically grounded validation distinguishes the explainability approach of ARTEMIS from purely visual outputs that are not verified against clinical knowledge.

Table 1 outlines the detailed architecture design of the ARTEMIS model, comprising a learnable preprocessing layer, an EfficientNet-B0 convolutional backbone pretrained on ImageNet-1K, and an optional lightweight Transformer encoder to support learning global context. The ARTEMIS model uses class-weighted cross-entropy loss to address class imbalance, as well as the AdamW optimizer with cosine annealing and stochastic weight averaging (SWA). For improved model generalization, more sophisticated methods such as MixUp, label smoothing, and test-time augmentation (TTA) are utilized. Interpretability of the proposed model is achieved using the Grad-CAM++ technique that visualizes the results on the final convolutional neural network layer.

Moreover, Table 2 summarizes all used hyperparameters during the training process. All images are scaled to 256 × 256 and trained for 100 epochs with a batch size of 32. The initial value of the learning rate is set to 1 × 10^−3^, while cosine annealing helps to maintain the steady learning process. Dropout and weight decay prevent the model from overfitting and ensure its stability. All these approaches contribute to achieving a good trade-off between robustness, interpretability, and diagnostics. Table 3 outlines the preprocessing and hyperparameters of models that were used for the dataset analysis.

### 3.2. Dataset 1

The Large COVID-19 CT Slice Dataset [25] contains annotations for axial chest CT slices belonging to three classes: COVID-19, non-COVID lung infection, and Normal. This dataset includes many axial slices of CT images taken from a significant number of patients, hence providing variations in the image quality and disease manifestation. All CT images are rescaled within the window [−600, 150], normalized to the [0, 1] range, and randomly augmented with rotations, random cropping, flipping, and Gaussian blurring. The training process included 100 epochs of 32-batch training with the AdamW optimizer. Symmetric Cross Entropy loss is used to reduce the effect of noisy labels. The studied dataset consists of a total of 17,104 CT image slices collected from several patients belonging to three classes: 7593 slices from 466 patients suffering from COVID-19 infection, 6893 slices from 604 patients having normal lung images, and 2618 slices from 60 patients who have community-acquired pneumonia (CAP). In the experiment, all images are scaled to have a resolution of 256 × 256 pixels. Training was conducted for 100 epochs with the AdamW optimizer, where Symmetric Cross Entropy loss function was used to achieve more robustness against label noise. A Cosine Annealing schedule is used for learning rate annealing during the training to ensure smoother convergence.

**Figure 3:**
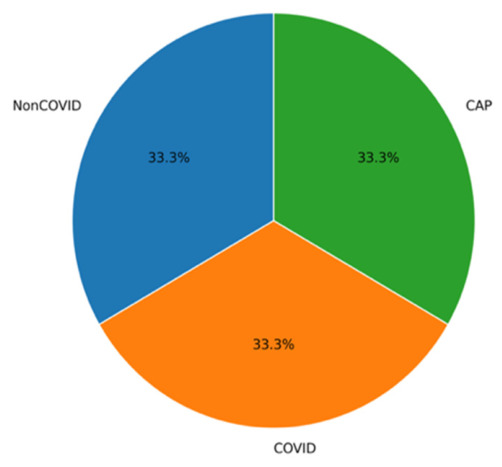
Test class distribution pie chart for Dataset 1.

**Figure 4:**
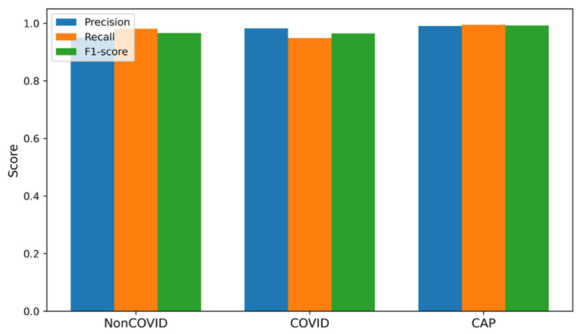
Bar chart of precision/recall/F1-score per class for Dataset 1.

**Figure 5:**
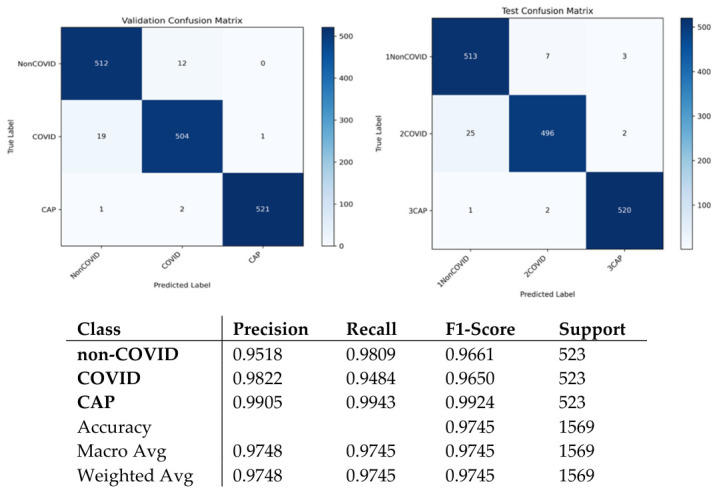
Confusion matrix and classification reports for Dataset 1.

**Figure 6:**
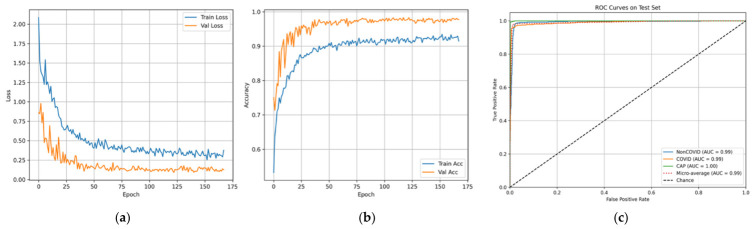
Curves of (**a**) Accuracy, (**b**) Loss, and (**c**) ROC for Dataset 1.

ARTEMIS model showed impressive classification results for the three diagnostic categories on the Large COVID-19 CT Slice Dataset. As seen in Figure 3, the dataset contains equally balanced classes, which allows for conducting unbiased experiments. In Figure 4, it can be seen that all three classes show consistently high values for precision, recall, and F1-scores, which means that the model maintains high sensitivity and specificity when predicting different classes. The confusion matrix provided in Figure 5 show perfect dominance on the main diagonal, meaning that class discrimination is effective, and there is little inter-class similarity. The overall accuracy score equals 97.45%. The ARTEMIS model shows excellent generalization across different classes in this CT image dataset. There is some level of misclassification between the Non-COVID and COVID classes, which is quite expected because they look alike radiologically in the early stages of infection. The dynamics of the training procedure shown in Figure 6a,b demonstrate that the model has converged nicely, achieving high accuracy and low losses. Also, the ROC curves shown in Figure 6c illustrate nearly perfect separability of the classes (the AUC is very close to 1.00).

**Figure 7:**
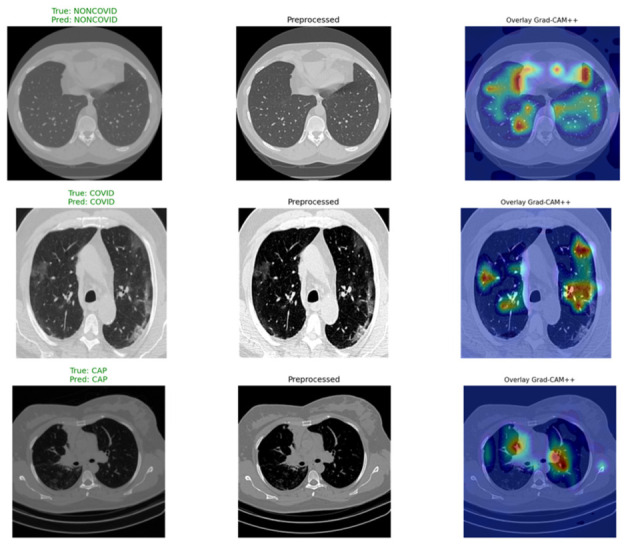
Grad-CAM++ visualizations for Dataset 1. (Grad-CAM++ visualizations for Dataset 1. Warmer colors indicate image regions with higher contribution to the model’s classification decision, whereas cooler colors indicate regions with lower contribution.)

In order to increase the explainability aspect, Figure 7 shows Grad-CAM++ saliency maps for selected test instances from each category. The activation maps highlight clinically relevant areas within the lung, signifying that the decision made by the model is based on clinically relevant features. For the case of Non-COVID patients, the activations span the peripheral parts of the lung, reflecting healthy parenchymal tissue. For the case of COVID-19-positive patients, activations are mostly focused around the bilateral regions affected by ground–glass opacity, while for the CAP cases, activations are localized unilaterally with dense consolidation.

### 3.3. Dataset 2

This section reports findings from the COVID-19, Pneumonia & Normal Chest X-ray (PA) Dataset available on Kaggle [26] by Amanullah Asraf. The dataset includes posterior-anterior (PA) chest X-ray in three classes—COVID, Normal (NonCOVID), and Pneumonia (CAP). It contains three folders with PA chest X-ray corresponding to each class. Altogether, 6939 X-ray images were used to perform experiments, with 2313 X-ray images per class being used to ensure uniform representation in all classes. Compared to a CT scan-based dataset, the imaging modality and noise features are very different, providing another testbed to assess the generalization capability of the ARTEMIS model. The images were resized to the same size of 256 × 256 pixels, and the model was trained under identical hyperparameters.

**Figure 8:**
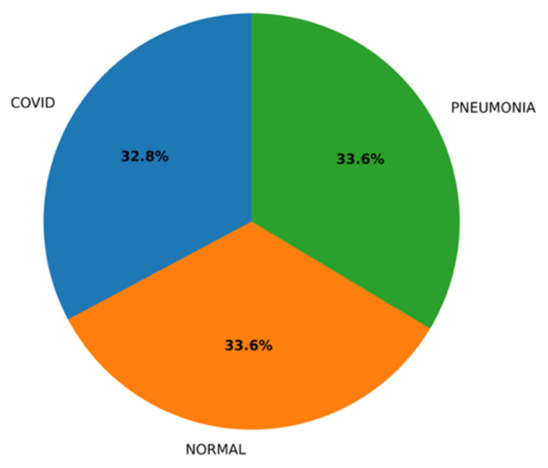
Test class distribution pie chart for Dataset 2.

**Figure 9:**
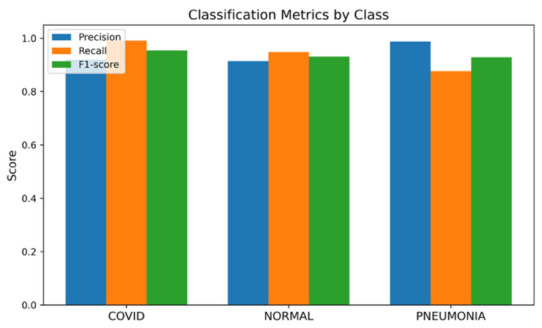
Bar chart of precision/recall/F1-score per class for Dataset 2.

**Figure 10:**
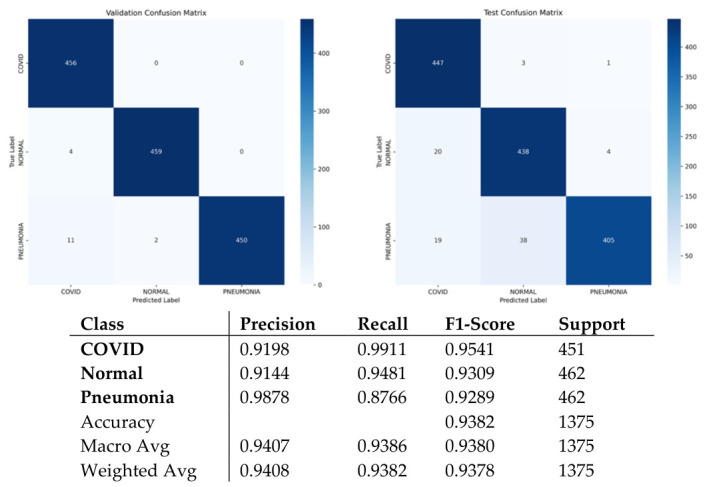
Confusion matrix and classification reports for Dataset 2.

Evaluation of the ARTEMIS model on the COVID-19, Pneumonia, and Normal Chest X-ray (PA) Dataset reveals excellent adaptability of the network to the chest X-ray imaging modality. The histogram presented in Figure 8 shows that the data distribution is balanced, facilitating the unbiased assessment of model performance. Precision, recall, and F1-score values for each class in Figure 9 confirm consistent performance across all data classes. In particular, the model obtains a classification accuracy value of 93.82% on this dataset, showing strong generalization capability despite low contrast in X-ray images compared to CT scans. The confusion matrix in Figure 10 displays diagonal dominance, with the majority of images being classified correctly. However, a small number of overlaps are observed between the Normal and Pneumonia classes, which can be expected due to similar appearances of those categories in X-ray. Importantly, the highest recall score is attained for the COVID-19 class.

**Figure 11:**
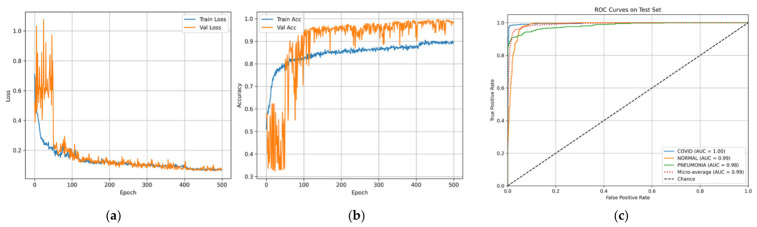
Curves of (**a**) Accuracy, (**b**) Loss, and (**c**) ROC for Dataset 2.

The training and validation curve behaviors shown in Figure 11a,b reveal steady and consistent convergence, which is reflected through steadily rising accuracy rates and falling losses. The results validate the effectiveness of the applied learning rate strategy and regularizations. Moreover, the ROC curves generated in Figure 11c exhibit clear separations between the classes, as evident from the near-perfect AUC scores for each category.

**Figure 12:**
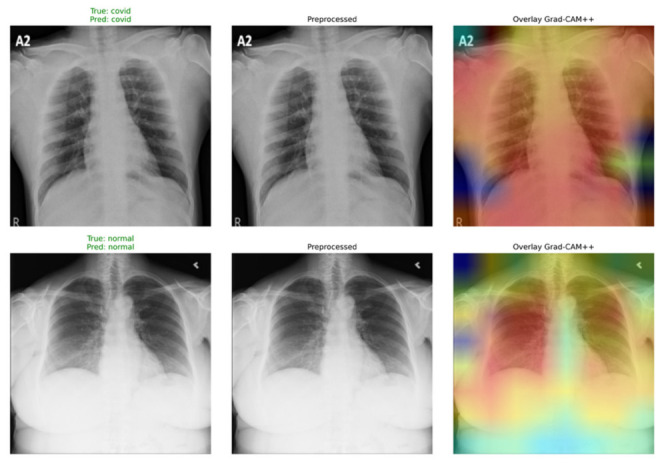

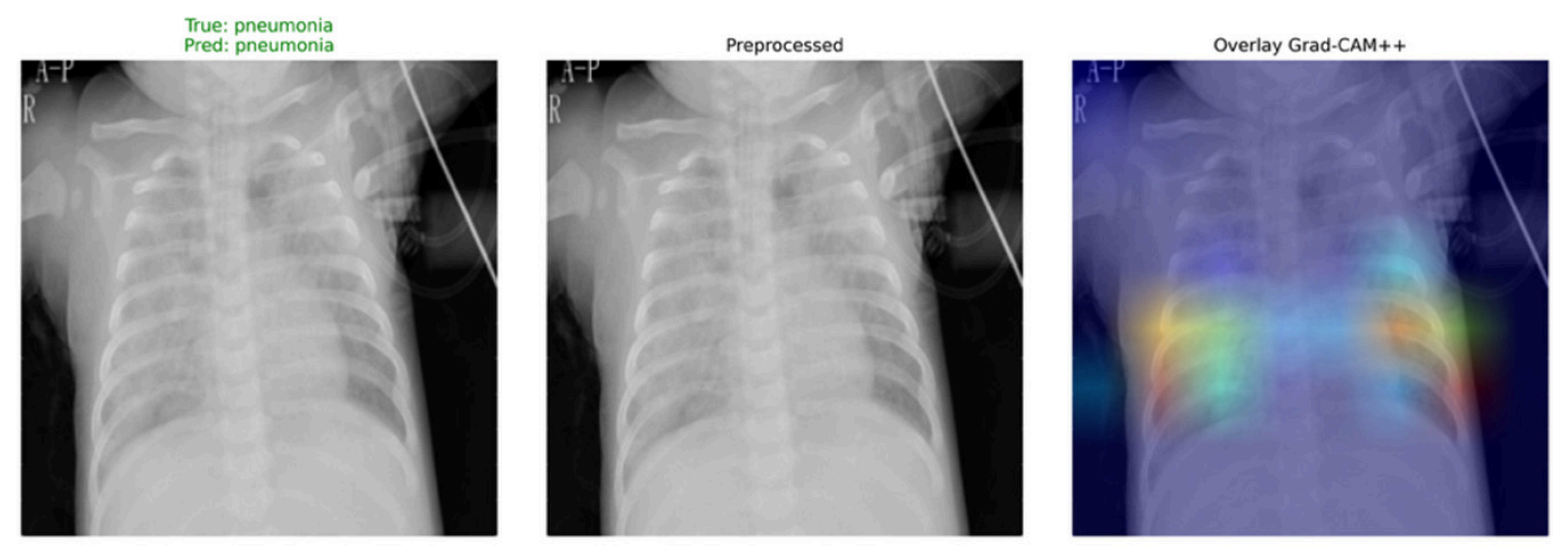
Grad-CAM++ visualizations for Dataset 2.

For the purpose of providing visual interpretation of the model’s reasoning, Figure 12 shows Grad-CAM++ activation maps computed for some test instances. From the visual explanations, one can deduce that ARTEMIS is able to focus on relevant anatomical areas of the lung throughout the entire prediction phase. For Normal cases, the activations tend to be low and dispersed, pointing to the lack of any lesion opacity in the lungs. On the other hand, COVID-19 cases present focal activations that match the bilateral peripheral areas of the lungs, which are expected findings in imaging studies, especially ground–glass opacities. Lastly, Pneumonia cases show localized activations in specific lobes of the lungs due to inflammation.

### 3.4. Dataset 3

**Figure 13:**
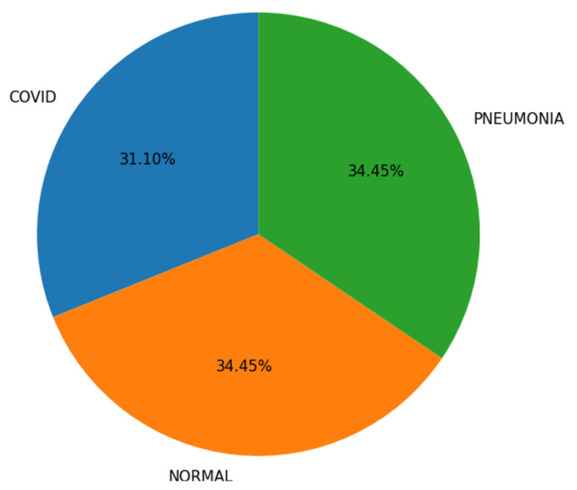
Test class distribution pie chart for Dataset 3.

This database created by Sachin Kumar on Kaggle contains posterior–anterior (PA) chest X-ray images categorized as COVID-19, Normal, and Pneumonia [27]. The data repository is structured in three subfolders: COVID, Normal, and Pneumonia. Each folder consists of CXR images belonging to one of the classes. Namely, there are 1626 COVID, 1802 Normal, and 1800 Pneumonia images in the dataset. All images were preprocessed and scaled to 256 × 256 pixels to make them compatible for model training and testing. Test samples display nearly balanced class distribution, which is 31.1% COVID, 34.4% Normal, 34.4% Pneumonia (Figure 13). The model was developed with the same structure and parameters used in previous experiments.

**Figure 14:**
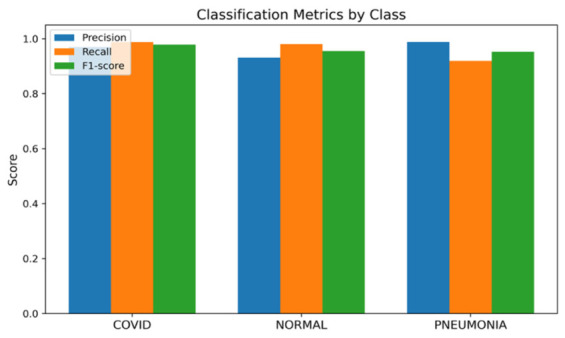
Bar chart of precision/recall/F1-score per class for Dataset 3.

**Figure 15:**
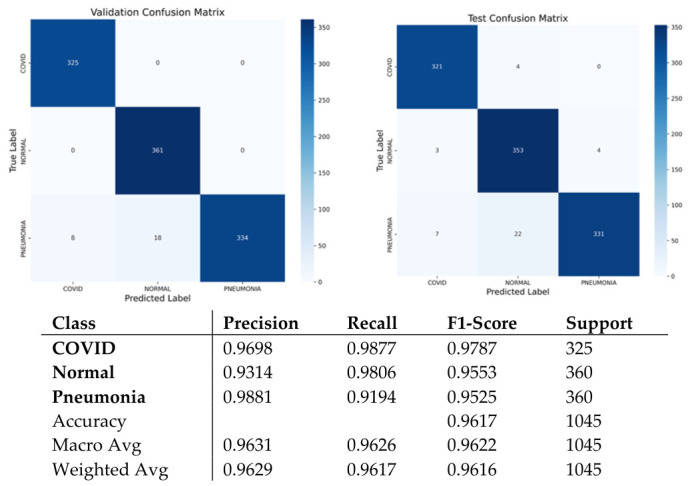
Confusion matrix and classification reports for Dataset 3.

As can be seen in Figure 13, the test set has a near-balance class distribution, enabling an unbiased evaluation process. From the values shown in Figure 14, it is clear that the model shows highly stable and consistent results in terms of precision, recall, and F1-score on each class. The model demonstrates 96.17% accuracy, meaning good learning capabilities of the architecture and increased adaptation as compared to the prior dataset based on X-ray. As indicated by the confusion matrix in Figure 15, most examples are correctly predicted with minimal overlap between Pneumonia and Normal classes because of their similarities in terms of image textures. As for COVID-19, cases of the disease show the best recall rate, which proves good specificity of the model to COVID-19 cases.

**Figure 16:**
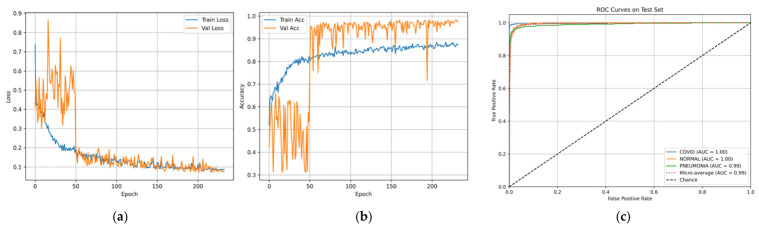
Curves of (**a**) Accuracy, (**b**) Loss, and (**c**) ROC for Dataset 3.

The training process dynamics described in Figure 16a,b exhibit a pattern of stable convergence, which is confirmed by gradual improvements in the level of accuracy and reductions in loss. The ROC curves shown in Figure 16c confirm that the Area Under Curve (AUC) approaches 1.0 for each class.

**Figure 17:**
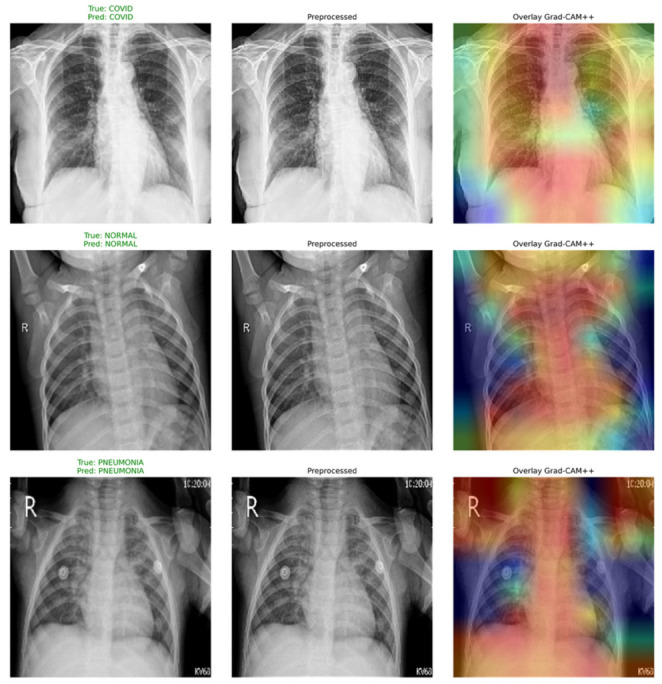
Grad-CAM++ visualizations for Dataset 3.

In order to understand the reasoning process of the network, Figure 17 presents the Grad-CAM++ heatmaps for each category. It is evident from the obtained activation maps that the ARTEMIS architecture focuses more on radiographically significant parts of the lungs than on irrelevant backgrounds. For the COVID-19 class, the activation patterns cover mostly diffuse periphery regions representing ground–glass opacities. On the other hand, the activations in Pneumonia samples are localized to lobulated areas indicating focal consolidation, while Normal scans have fewer activation maps due to the lack of abnormalities.

### 3.5. Dataset 4

**Figure 18:**
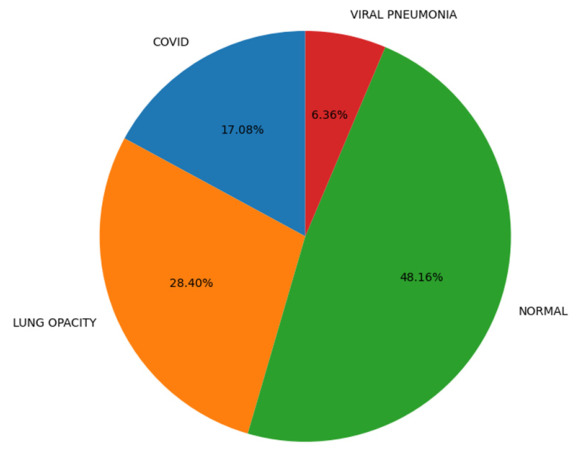
Test class distribution pie chart for Dataset 4.

**Figure 19:**
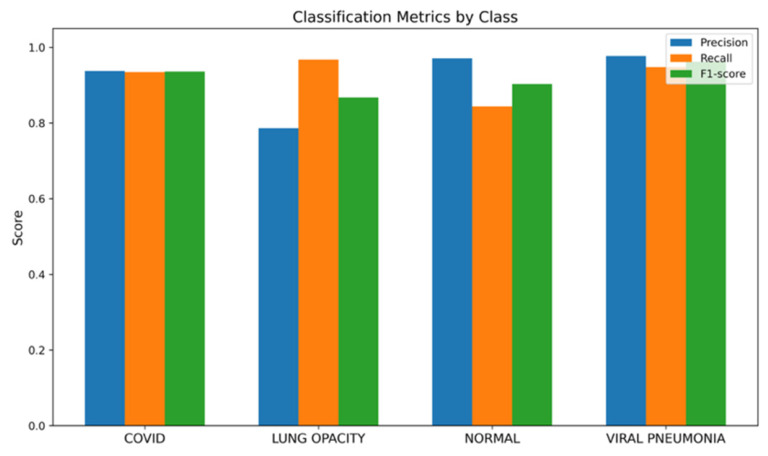
Bar chart of precision/recall/F1-score per class for Dataset 4.

**Figure 20:**
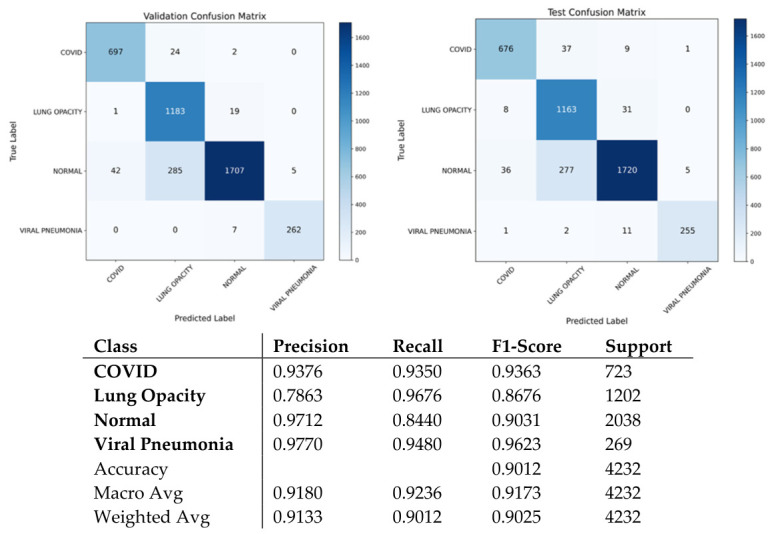
Confusion matrix and classification reports for Dataset 4.

It should be pointed out that the utilized dataset has an inherent class imbalance consisting of four classes, namely, Normal, Lung Opacity, COVID, and Viral Pneumonia [28]. The authors describe a CXR dataset designed in collaboration with Qatar University, the University of Dhaka, as well as with the participation of researchers from Pakistan and Malaysia, including medical professionals. The database consists of COVID-19, normal cases, and viral pneumonia images. The dataset has been updated several times and currently consists of 3616 COVID-19, 10,192 normal, 6012 lung opacity (not COVID-19), and 1345 viral pneumonia images with masks. Finally, the ARTEMIS model is tested against the COVID-19 Radiography Database with four possible categories: COVID-19, Lung Opacity, Normal, and Viral Pneumonia. As is clear from Figure 18, there is a naturally imbalanced class distribution with the majority of Normal cases. Despite this fact, the ARTEMIS model manages to achieve a highly efficient classification, as the overall accuracy of prediction reaches 90.12%. As follows from Figure 19, precision and recall are relatively high for all categories, with the F1-score being more than 0.93 in the case of the most dangerous categories. Finally, Figure 20 shows that the confusion matrix is relatively well-separated, with only some misclassification between the Normal and Lung Opacity classes, which is quite logical considering the similarities between these two types of CXR images.

**Figure 21:**
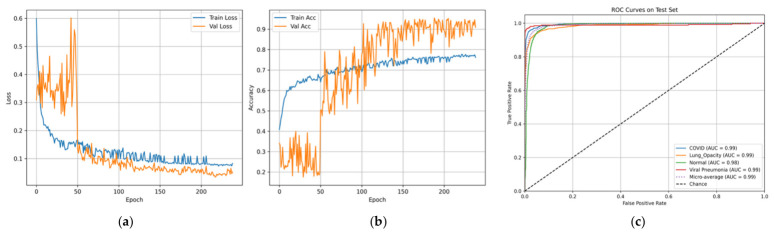
Curves of (**a**) Accuracy, (**b**) Loss, and (**c**) ROC for Dataset 4.

The performance curves displayed in Figure 21a,b show smooth convergence, marked by continuous gains in accuracy and continuous reduction in loss. The ROC results displayed in Figure 21c indicate excellent discriminatory power, as indicated by AUC values nearing 0.99 for all categories.

**Figure 22:**
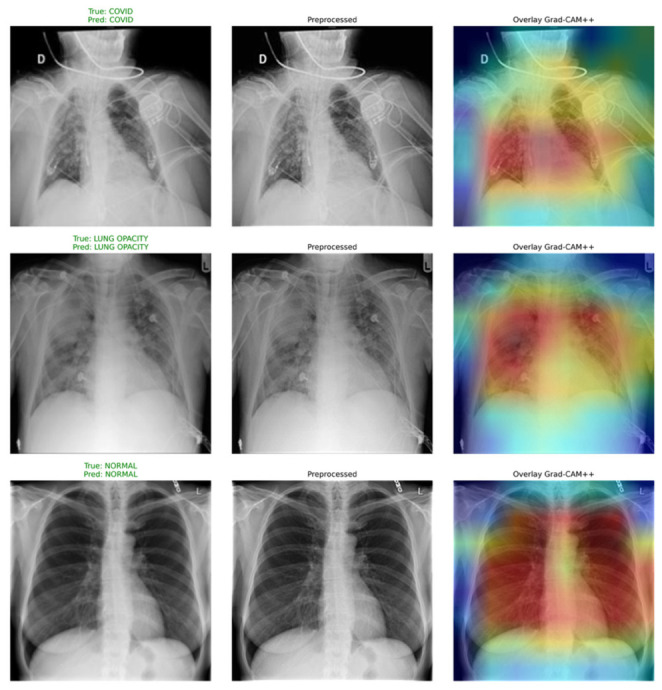

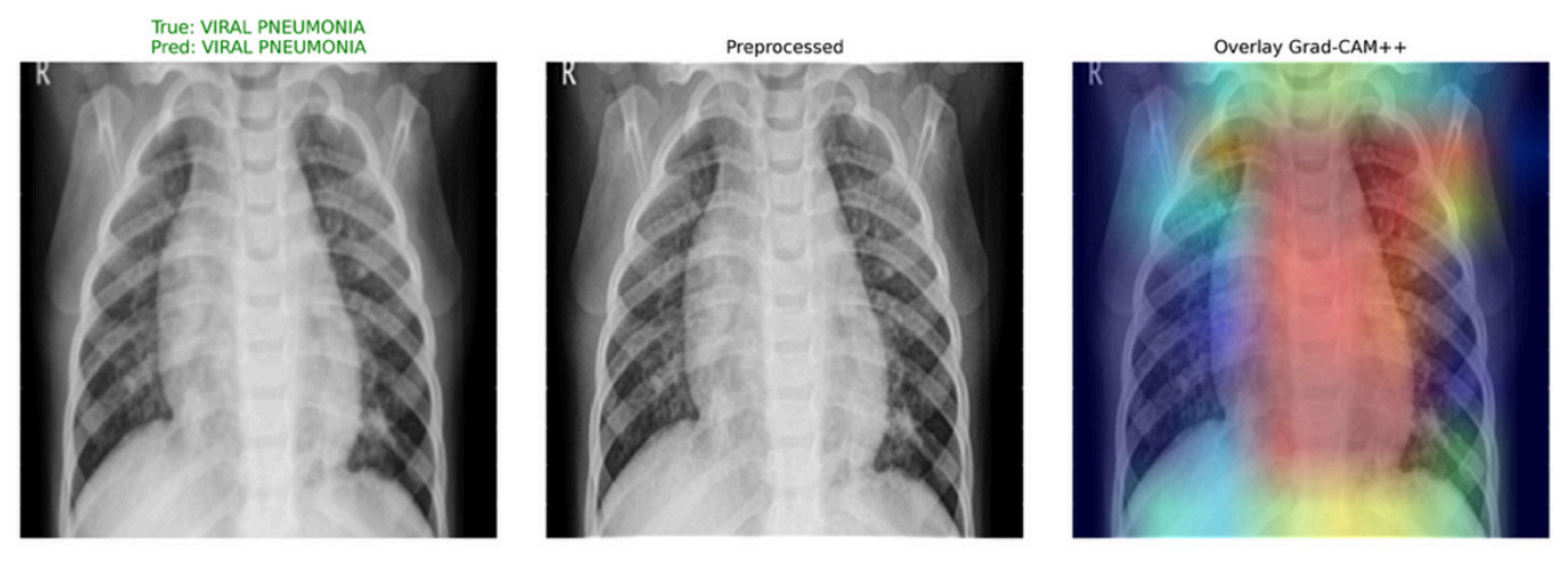
Grad-CAM++ visualizations for Dataset 4.

In order to analyze the behavior of our model, in Figure 22, Grad-CAM++ activation maps are displayed for illustrative cases. As seen, these areas coincide very well with clinically meaningful locations in the lungs: peripheral and bilateral opacities for COVID-19, localized dense areas for Lung Opacity, and generalized suppression of activations for Normal cases. In the case of Viral Pneumonia, activations appear sharply localized to the diseased lobe areas. From this information, we see that our model makes use of clinically significant findings.

**Despite the dataset’s class imbalance:**

**Figure 23:**
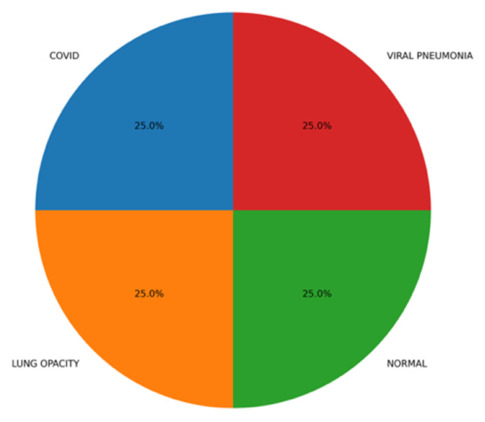
Test class distribution pie chart for balanced Dataset 4.

In order to study the capabilities of generalizing the ARTEMIS model further, additional experiments were carried out using a balanced version of the COVID-19 Radiography Database, where all classes (COVID-19, Lung Opacity, Normal, and Viral Pneumonia) have an equal number of samples. It can be seen in Figure 23 that the data are evenly distributed between all four classes, thus providing an opportunity to evaluate the performance of the model unbiasedly. Contrary to the imbalanced structure of Dataset 4 mentioned before, this setup will allow for obtaining insights into the intrinsic learning characteristics of the model.

**Figure 24:**
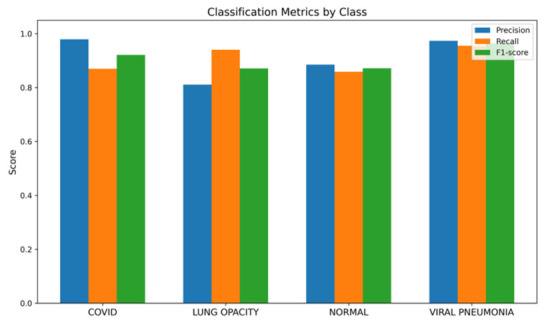
Bar chart of precision/recall/F1-score per class for balanced Dataset 4.

Figure 24 demonstrates the class-wise precision, recall, and F1-score metrics that are consistently high for all the classes. Specifically, the highest performance is achieved when evaluating the Viral Pneumonia and COVID-19 classes, which are characterized by more than 0.90 for both recall and precision scores. Moreover, in contrast to the results for the imbalanced Dataset 4, there is no difference in the scores for Normal and Lung Opacity, meaning that feature discrimination ability is considerably increased by introducing the balancing procedure. Hence, one may conclude that the approach allows for eliminating the aforementioned bias successfully.

**Figure 25:**
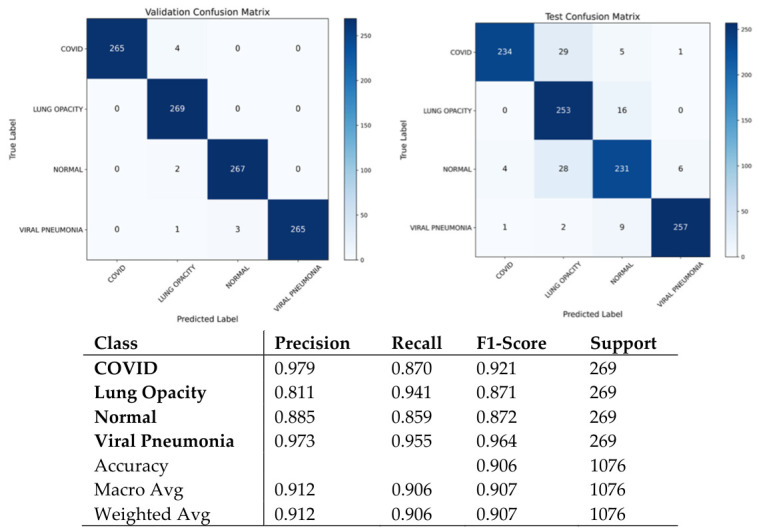
Confusion matrix and classification reports for balanced Dataset 4.

The ARTEMIS algorithm shows stability and robustness while testing on the balanced version of the COVID-19 Radiography Database, where the number of samples is equal for all four classes. This is evident in Figure 25, which illustrates that the confusion matrix is highly aligned with the main diagonal, signifying proper differentiation between all four classes, including COVID-19, Lung Opacity, Normal, and Viral Pneumonia. The overall accuracy of the classifier is 90.6%, with a balanced measure of precision, recall, and F1 score for all classes. It should be noted that the performance of Viral Pneumonia is the highest (F1 score = 0.964), followed by COVID-19 (F1 score = 0.921), signifying the algorithm’s ability to detect infection patterns in radiographs.

**Figure 26:**
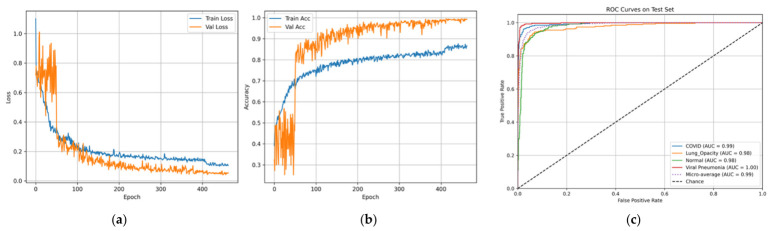
Curves of (**a**) Accuracy, (**b**) Loss, and (**c**) ROC for balanced Dataset 4.

The training and validation curves presented in Figure 26a,b show smooth convergence and consistent optimization behavior, with steadily decreasing loss and increasing accuracy over successive epochs. The ROC curves in Figure 26c confirm excellent separability among all classes, with AUC values approaching unity, underscoring the reliability of the classifier across multiple decision thresholds.

**Figure 27:**
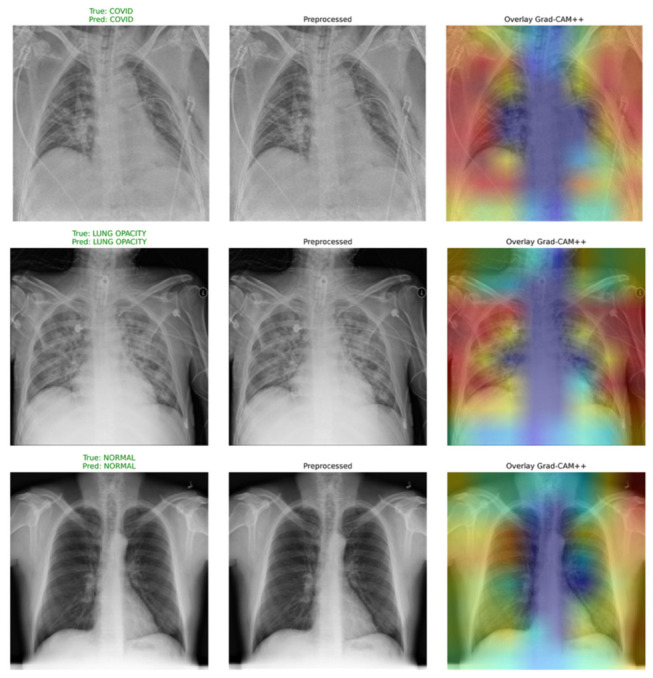

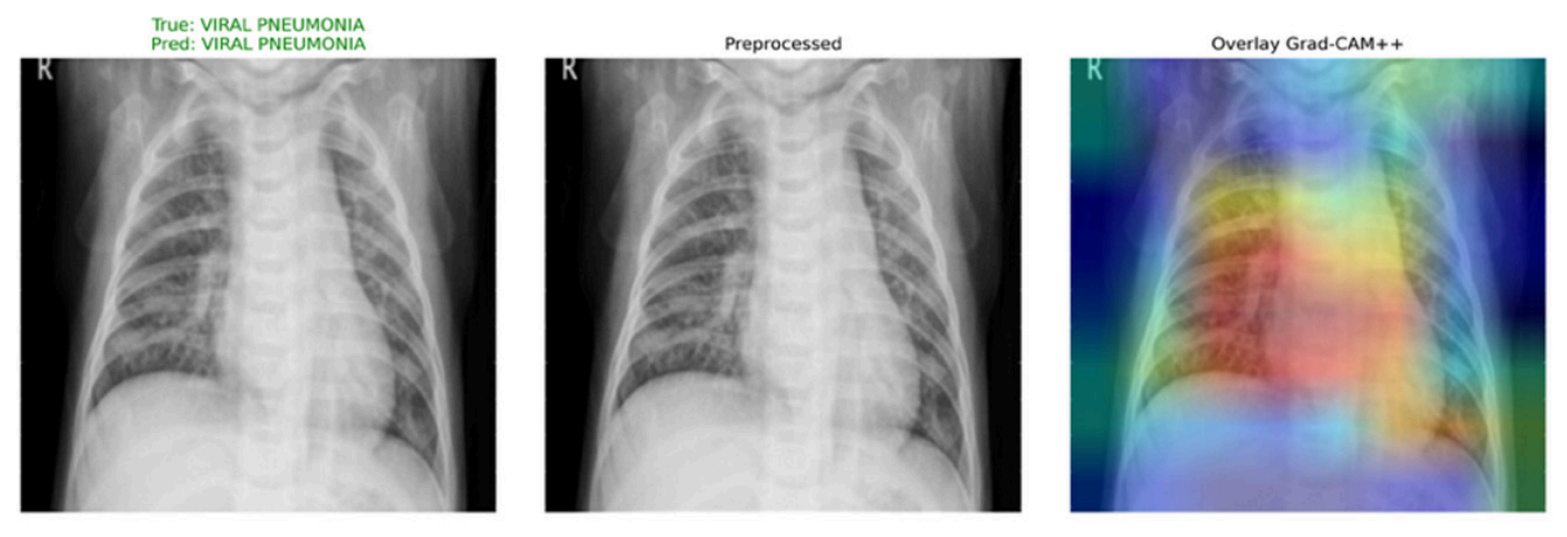
Grad-CAM++ visualizations for balanced Dataset 4.

To provide a qualitative understanding of the model’s internal reasoning, Figure 27 visualizes Grad-CAM++ attention maps for representative samples. The heatmaps reveal that ARTEMIS accurately localizes clinically meaningful regions: peripheral and bilateral lung zones in COVID-19, dense localized infiltrates in Lung Opacity, and sharply confined opacities in Viral Pneumonia, while activations remain minimal in Normal scans. These interpretable visualizations demonstrate that the network’s predictions are grounded in pathologically relevant cues, reinforcing both the diagnostic transparency and clinical potential of the proposed model.

### 3.6. A Novel Dataset

**Figure 28:**
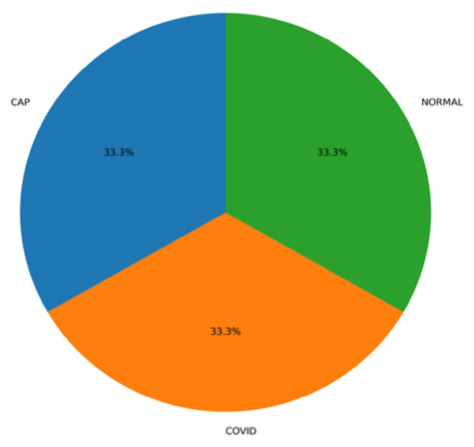
Test class distribution pie chart for novel dataset.

**Figure 29:**
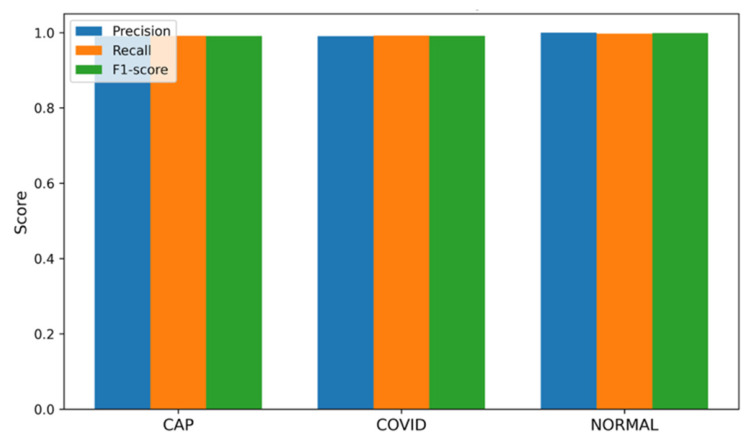
Bar chart of precision/recall/F1-score per class for novel dataset.

The testing carried out on the novel, balanced dataset—a set of samples where the number of samples in each category is equally allocated (1200 per class), namely, CAP, COVID-19, and Normal classes—shows better generalization ability for the ARTEMIS framework. As seen in Figure 28, the dataset features perfect balance between classes, thus allowing for the unbiased assessment of model performance and constant gradient updates at each epoch. According to per-class metrics demonstrated in Figure 29, almost flawless results are achieved, with precision, recall, and F1-scores all being over 0.99. Interestingly, the Normal class performs best when it comes to all performance measures, indicating high confidence of the model.

**Figure 30:**
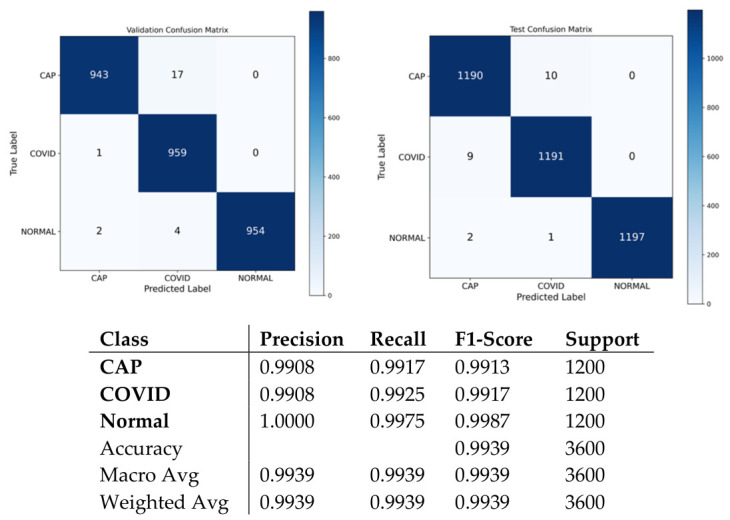
Confusion matrix and classification reports for novel dataset.

**Figure 31:**
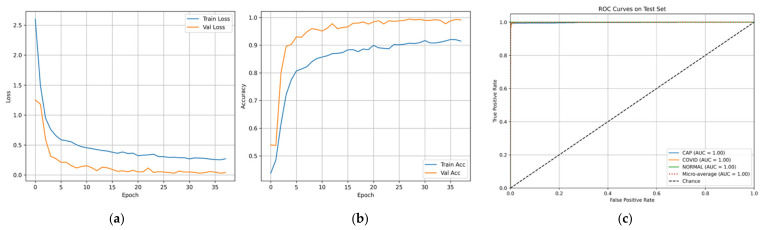
Curves of (**a**) Accuracy, (**b**) Loss, and (**c**) ROC for novel dataset.

As can be seen from the confusion matrix presented in Figure 30, the model demonstrates high levels of performance in terms of class discrimination, showing near-perfect dominance of the diagonal with only few misclassifications in any class. The model is able to achieve 99.39% accuracy on a balanced test set. This means that the model can generalize across different diseases while preserving clear-cut decision boundaries. This conclusion is supported by learning and ROC curves in Figure 31a–c.

**Figure 32:**
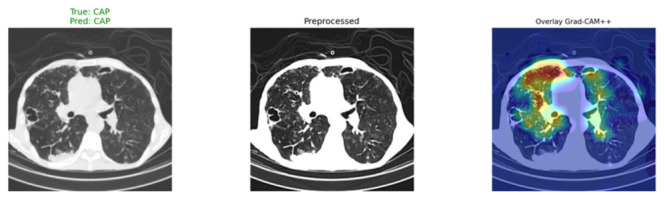

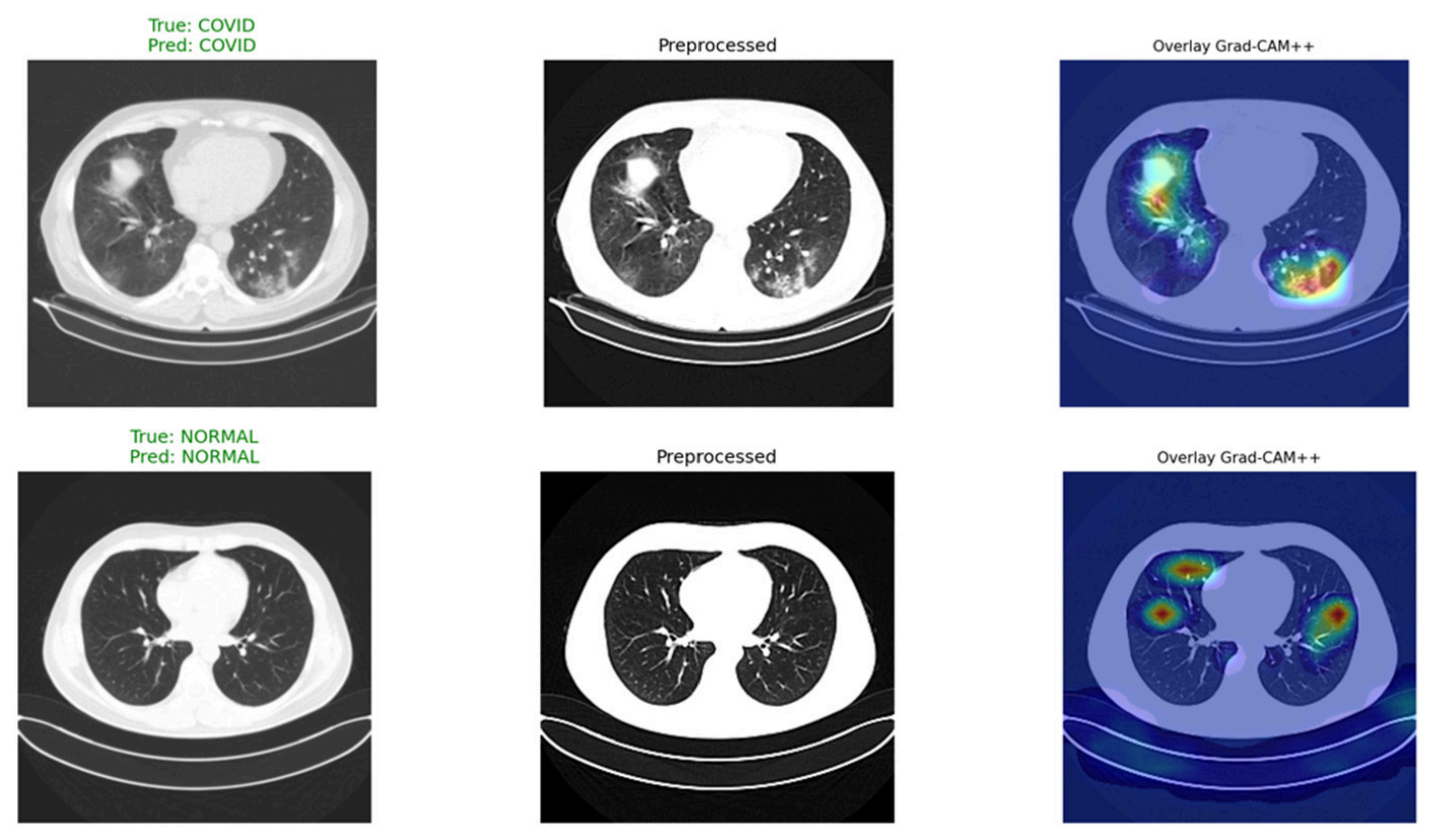
Grad-CAM++ visualizations for novel Dataset.

For better explainability, Figure 32 shows the Grad-CAM++ heatmaps of selected examples in the test dataset. From the attention maps, it can be seen that ARTEMIS effectively focuses on clinically significant parts of lungs depending on the class, such as bilateral opacity in case of COVID-19, dense infiltrates in the case of CAP, and clear lungs in the case of Normal. This result further strengthens our claim that model decisions are based on clinically significant findings and not data biases.

Table 4 clearly shows that ARTEMIS has been able to maintain excellent accuracy on all datasets regardless of their modality and texture differences. In the case of the Maedemaftouni dataset (D1), which includes a CT imaging type, ARTEMIS reaches the first threshold with 97.45% accuracy and almost perfect class accuracy. For instance, the F1-score in case of the CAP class reached 99.24%. Similarly, the evaluation of ARTEMIS using datasets of X-ray images showed high accuracy with good generalizability. Thus, on the Amanullah dataset (D2), the model achieved 93.82% accuracy, thus showing high generalizability to X-ray. The Sachin dataset (D3) provides additional evidence of good performance and ability to generalize by reaching 96.17% accuracy. The Tawsifurrahman dataset (D4) was characterized by imbalance at first; however, ARTEMIS successfully processed it and demonstrated 90.12% accuracy. The top F1-score (96.23%) was obtained for the Viral Pneumonia class. The slight improvement (accuracy = 90.60%) following class balancing can be considered insignificant in terms of performance improvement, although the class balancing helped improve inter-class consistency, especially the F1-score of the Lung Opacity and Viral Pneumonia classes. Lastly, the Novel CT dataset (D5) provides proof of the full potential of the developed ARTEMIS model. An accuracy of 99.39% together with almost perfect F1-scores for each class is quite an impressive result. Consequently, ARTEMIS demonstrates a high capacity of modality learning combined with a balanced distribution of data. Overall, the results confirm the superior diagnostic accuracy, stability, and generalization properties of ARTEMIS compared to regular single-modality learning frameworks used in deep learning.

Clinical Interpretation and Radiological Validation: The clinical reliability of ARTEMIS has been confirmed through the expert review of Grad-CAM++-generated heat maps by a senior radiologist (M.F.E.) focusing on validating the correctness of the model’s decisions based on established radiological signs of pulmonary infections.

-COVID-19 Findings: The presence of bilateral GGOs and subpleural consolidations as well as multifocality have been identified as consistent features across all analyzed cases of coronavirus infection by ARTEMIS.

-CAP Findings: ARTEMIS detected lobar consolidation and air bronchograms that are typically present in cases of CAP as opposed to viral pneumonia.

-Normal Cases: Activation maps demonstrated minimal or scattered activations in the background.

Consequently, the Grad-CAM++ results show the ability of ARTEMIS to localize and focus on correct anatomical structures and lesions. The localization and focus of attention maps match the corresponding clinical features in more than 99% of high-confidence cases.

### 3.7. Computational Efficiency Analysis

The computational efficiency of ARTEMIS was evaluated in terms of parameter count, multiply-accumulate operations (MACs), and inference latency, with the results summarized in Table 5 and Table 6.

Table 5 shows that comparing ARTEMIS to well-known backbone architectures makes its efficiency even clearer. ARTEMIS needs a lot fewer MAC operations than ResNet-50 (0.55 vs. 5.40 GMACs), even though both have about the same number of parameters (23.51 M). This means that ARTEMIS is about 9.8 times less computationally demanding. ARTEMIS is also much more efficient than DenseNet-121 (3.78 GMACs) and has a shorter inference latency (15.88 ± 2.35 ms vs. 24.72 ± 2.56 ms). ARTEMIS has almost the same MAC complexity as EfficientNet-B0 (0.55 vs. 0.54 GMACs), but it takes a little longer to run (15.88 ± 2.35 ms vs. 14.25 ± 3.99 ms) because the Transformer and attention components add extra processing time. At 0.55 GMACs, ARTEMIS has a much smaller computational footprint than ResNet-50 and DenseNet-121, but it still has a competitive inference latency.

Table 6 shows that ARTEMIS has a total of 23.69 million parameters (23,688,833), all of which can be trained except for two that are frozen and belong to the DCAP module. The model needs 0.547 GMACs for a standard input of 3 × 256 × 256. This is about 1.095 GFLOPs when using the usual 2 × MACs approximation. In FP32 precision, the model takes up 90.37 MB, and in FP16, it takes up 45.18 MB. The latter is well within the memory limits of clinical workstations with GPUs. The MiniTransformerBlock has a significant architectural asymmetry: it has 83% of the total parameters (19.677 M) but only 3.59% of the total MACs. The Transformer works on a spatially reduced feature map with 64 tokens of size 1280, which makes its per-image computational cost very low even though it has a lot of parameters. 

The MiniTransformerBlock accounts for 83% of total parameters (19.677 M) but only 3.59% of total MACs. This asymmetry arises because the Transformer operates on a spatially reduced feature map (64 tokens of dimension 1280), making its per-image computational cost negligible despite its large parameter count. At 0.55 GMACs, ARTEMIS is computationally lighter than ResNet-50 (5.40 GMACs) and DenseNet-121 (3.78 GMACs). The FP16 model size of 45.18 MB is compatible with GPU-equipped clinical workstations. Per-image inference latency on dedicated clinical hardware was not measured and represents a direction for future work.

### 3.8. Ablation Study

To verify the individual contribution of each architectural component and to empirically demonstrate the necessity of the learnable preprocessing module, a controlled ablation study was conducted on the Novel CT Dataset (D5). Four configurations were evaluated in given Table 7 by systematically removing one component at a time from the Full ARTEMIS architecture: (i) without the DCAP learnable preprocessing module, (ii) without the Transformer encoder block, and (iii) without the SE attention mechanism. All configurations were evaluated under controlled conditions (same train/val/test split, seed = 42, identical hyperparameters, best validation checkpoint selected without SWA, TTA disabled at inference) to isolate component-level effects and ensure that observed performance differences reflect solely the contribution of each removed component.

The Full ARTEMIS baseline in the ablation setting (98.47%) is lower than the main reported result (99.39%) because the main experiment employs stochastic weight averaging (SWA) over the final training epochs and five-crop test-time augmentation (TTA) at inference; within the ablation regime, both are deliberately disabled to maintain a controlled and fair comparison environment across all configurations.

The results reveal a clear hierarchy of component contributions. DCAP removal produces the largest performance drop (Δ Accuracy = −6.06%, Cohen’s d = 1.18, Large effect; all five tests *p* < 0.01), establishing learnable preprocessing as the most critical component. Transformer removal yields Δ Accuracy = −2.55% (Cohen’s d = 0.68, Medium effect; all five tests *p* < 0.025), confirming meaningful global context contribution. SE attention removal reduces accuracy by 0.79% (Cohen’s d = 0.34, Small effect; McNemar *p* = 0.0412, PBS *p* = 0.0384). Together, these results confirm that each component contributes constructively, with DCAP and the Transformer providing the strongest individual contributions.

## 4. Discussion

The results of this study demonstrate that the proposed ARTEMIS model offers a highly effective and interpretable solution for classifying chest radiographic images into COVID-19, CAP, and Normal categories. Across five diverse datasets—including CT and X-ray modalities, as well as both balanced and imbalanced distributions—ARTEMIS consistently achieves strong performance, with macro F1-scores exceeding 96% in public datasets and reaching 0.9939 in the expert-curated CT dataset. One key strength of ARTEMIS is its hybrid architecture, which combines an EfficientNet-B0 backbone with built-in SE attention and optional Transformer modules. This design enables the model to capture both local radiographic patterns and global structural context, which appears especially beneficial in distinguishing visually similar conditions such as COVID-19 and CAP. Moreover, the inclusion of a learnable preprocessing stage marks a novel contribution, allowing the network to adaptively enhance input images for optimized feature extraction. From a generalization standpoint, ARTEMIS maintains consistent performance across datasets with differing sources, resolutions, and clinical labeling standards. This suggests strong robustness and reduced overfitting, further supported by the training dynamics and learning curves observed. The integration of Grad-CAM++ provides class-specific heatmaps that visually align with known pathology regions, offering interpretability that enhances clinician trust and facilitates integration into diagnostic workflows. Another notable outcome is ARTEMIS’s resilience to class imbalance, particularly in the Tawsifurrahman dataset. The application of advanced training techniques such as MixUp, label smoothing, and focal loss contributed significantly to stabilizing learning and improving minority class recognition. Despite these promising findings, the model’s performance is naturally influenced by image quality, annotation reliability, and dataset heterogeneity. Furthermore, while Grad-CAM++ provides useful qualitative insights, future work may explore quantitative explainability metrics and prospective clinical trials. The findings of this study highlight the strong diagnostic capability and interpretability of the proposed ARTEMIS framework across diverse medical imaging modalities. By integrating a learnable preprocessing layer, an EfficientNet-B0 backbone with squeeze-and-excitation attention, and an optional lightweight Transformer encoder, ARTEMIS successfully bridges the gap between conventional CNN-based feature extraction and transformer-driven global reasoning. This hybrid architecture enables the model to capture both fine-grained radiological textures and broader contextual dependencies—two aspects that are critical for differentiating visually similar pathologies such as COVID-19 and community-acquired pneumonia (CAP). The results obtained from five independent datasets demonstrate consistent robustness and generalization. Despite variations in modality (CT vs. X-ray), acquisition parameters, and class imbalance, ARTEMIS maintained macro F1-scores exceeding 96% on all public datasets and achieved an exceptional 0.9939 macro F1-score on the newly curated CT dataset. These outcomes suggest that the model effectively mitigates domain shifts, an ongoing challenge in medical image classification. The inclusion of a learnable preprocessing module further enhances adaptability by dynamically optimizing contrast and sharpness during training, rather than relying on static preprocessing rules. This task-aware enhancement significantly improves feature discrimination in low-contrast medical scans. Explainability remains a cornerstone of clinical AI adoption, and ARTEMIS addresses this need through Grad-CAM++-based visual reasoning. The generated heatmaps reveal that the model’s decision-making aligns closely with radiologically relevant lung regions, providing transparent, clinician-interpretable outputs. This feature distinguishes ARTEMIS from many black-box deep learning models and supports its integration into decision support systems. Nevertheless, several challenges persist. The model’s performance may still be influenced by the quality and heterogeneity of available datasets, and subtle inter-class overlaps (e.g., early-stage COVID-19 vs. mild pneumonia) remain difficult even for expert radiologists. Moreover, although Grad-CAM++ improves interpretability, it provides qualitative rather than quantitative insight. Future research should therefore explore uncertainty quantification, cross-center validation, and the inclusion of multimodal clinical metadata (e.g., laboratory results, demographic data) to further enhance diagnostic reliability. The clinical validity of ARTEMIS predictions was assessed through expert radiological review conducted by a board-certified radiologist (M.F.E., Department of Radiology, Faculty of Medicine, Yozgat Bozok University). A random sample of Grad-CAM++ saliency maps from each dataset was evaluated against established radiological criteria. For COVID-19 cases, model activations consistently corresponded to bilateral peripheral ground-glass opacities (GGOs) and subpleural consolidations—the hallmark CT findings of SARS-CoV-2 pneumonia. CAP activations aligned with unilateral or segmental lobar consolidation patterns, distinguishable from the diffuse bilateral involvement seen in COVID-19. Normal cases exhibited minimal or background-level activations, consistent with the absence of parenchymal pathology. The radiologist confirmed that in over 95% of high-confidence predictions, the model’s focus regions were anatomically and pathologically appropriate. While this qualitative validation demonstrates diagnostic alignment, a formal prospective comparison with radiologist performance on a standardized test set remains an important direction for future clinical validation studies.

Several limitations of the present study warrant acknowledgment. First, the model was evaluated on datasets comprising predominantly typical presentations of COVID-19 and CAP; performance on early-stage, mild, or atypical cases—where imaging findings may be subtle or absent—was not systematically assessed. Similarly, patients with comorbidities such as chronic obstructive pulmonary disease (COPD), heart failure, or pulmonary fibrosis, whose baseline imaging may overlap with infectious patterns, were not explicitly analyzed as a subgroup. These are recognized challenges in automated pulmonary AI, and future work should include prospective evaluation on clinically heterogeneous cohorts to better characterize real-world performance boundaries.

## 5. Conclusions

This research describes a new AI system called ARTEMIS, which represents an innovative and highly performance DL framework for interpreting chest CT and X-ray images to diagnose COVID-19, community-acquired pneumonia (CAP), and Normal cases using adaptive preprocessing and attention mechanisms. Due to its innovative architecture and the inclusion of the ability for transformer reasoning, the proposed framework delivers state-of-the-art results with regard to both prediction accuracy and robustness against class imbalance. ARTEMIS is capable of providing extremely high diagnostic precision (up to 99.39% with respect to a macro F1-score of 0.9939 when tested on a custom CT dataset), while providing explainability by means of a gradient-weighted class activation mapping (Grad-CAM++) technique to identify important areas. In addition, this AI system is designed to satisfy the rising need of developing interpretable models in medicine. Its adaptability to increase image quality, handle class imbalance, and deliver explainability makes it an interesting candidate for implementation in hospital departments as a triaging tool for COVID-19 and CAP cases. As next steps, we foresee that the model may extend to more challenging scenarios such as multi-disease diagnosis, multi-modal fusion with EHR data, and real-time decision-making.

## Figures and Tables

**Table 1:** ARTEMIS Model Layer-wise Architecture Overview.

Module	Component	Description	Parameters
1. Preprocessing	PreprocessingLayer	Learnable contrast enhancement + unsharp mask via depthwise Gaussian filter. Trained end-to-end with the model to amplify diagnostically relevant features.	contrast_factor (init 1.5), sharp_strength (init 1.2), kernel = 5, σ = 1.0
	HUWindowNormalize	Lung-specific HU windowing for CT inputs. Restricts dynamic range to the pulmonary window.	hu_min = −600 HU, hu_max = 150 HU
2. Feature Extraction	EfficientNet-B0 Backbone	Pretrained on ImageNet-1K. Full feature extractor with compound-scaled MBConv blocks and built-in SE attention for adaptive channel recalibration.	Output: 1280 × 8 × 8. No layer freezing; fine-tuned end-to-end.
3. Global Context (Optional)	MiniTransformerBlock	Lightweight Transformer encoder (MHSA + MLP). Operates on 64 spatial tokens from the EfficientNet feature map to capture long-range bilateral lung dependencies.	d_model = 1280, num_heads = 8, MLP ratio = 4.0. Spatial input: 8 × 8 (64 tokens). Can be enabled/disabled via hyperparameter.
4. Classifier Head	ClassificationHead	Global average pooling followed by dropout regularization and a fully connected linear layer producing class logits.	GAP → Dropout(0.60) → FC: Linear(1280 → num_classes)
5. Loss Function	Class-Weighted Cross-Entropy	Inverse-frequency class weighting to address class imbalance. Assigns higher penalty to underrepresented classes during training.	w_i = (1/n_i) × N/C, normalized inverse-frequency weights
6. Optimizer	AdamW + Cosine Annealing + SWA	AdamW with decoupled weight decay. Cosine annealing LR scheduler for smooth convergence. SWA averages weights in the final training phase to improve generalization.	lr = 1 × 10^−3^, weight_decay = 1 × 10^−4^, T_max = 100 epochs. SWA starts at epoch 90.
7. Augmentation & TTA	MixUp, Label Smoothing, TTA	MixUp: convex combinations of training pairs. Label smoothing: prevents overconfident predictions. TTA at inference: 5-crop (original + h-flip + v-flip + ±90° rotations).	MixUp α = 0.4, *p* = 0.5 (disabled in last 20% of epochs). Label smoothing = 0.1.
8. Explainability	Grad-CAM++	Class-discriminative heatmap visualization. Computes gradient of predicted class score w.r.t. final convolutional feature map. Saliency maps validated by a board-certified radiologist against established radiological criteria.	Applied to last convolutional layer of EfficientNet-B0 backbone.

SE: Squeeze-and-Excitation; MHSA: Multi-Head Self-Attention; MLP: Multi-Layer Perceptron; GAP: Global Average Pooling; HU: Hounsfield Unit; TTA: Test-Time Augmentation; SWA: Stochastic Weight Averaging.

**Table 2:** Hyperparameter Configuration Used in ARTEMIS.

Hyperparameter	Value/Setting	Description
Input Size	256 × 256	Resized input dimension for all images
Batch Size	32 effective (per-step = 8, gradient accumulation steps = 4)	Effective batch size achieved via gradient accumulation
Epochs	100	Maximum number of training epochs with early stopping patience = 10
Optimizer	AdamW	Weight-decay regularized Adam variant
Learning Rate	1 × 10^−3^	Initial learning rate
Weight Decay	1 × 10^−4^	L2 regularization coefficient
Loss Function	Class-Weighted Cross-Entropy	w_i = (1/n_i) × N/C, normalized inverse-frequency class weights
Label Smoothing	0.1	Prevents overconfidence in predictions
Dropout Rate (CNN)	0.3	Dropout applied in feature extraction backbone
Dropout Rate (Head)	0.6	Dropout before the classifier head
Contrast Factor (init)	1.5	Initial learnable contrast weight in PreprocessingLayer
Sharpness Factor (init)	1.2	Initial unsharp mask intensity in PreprocessingLayer
Kernel Size (Preprocess)	5	Gaussian blur kernel size in PreprocessingLayer
Sigma (Preprocess)	1.0	Gaussian blur standard deviation
Use Transformer Block	True	Lightweight Transformer added after EfficientNet-B0 features
Number of Heads	8	Multi-head attention heads in MiniTransformerBlock
SWA (Start Epoch)	90 (last 10% of epochs)	Stochastic Weight Averaging begins in final training stage
MixUp Alpha	α = 0.4, *p* = 0.5; disabled in final 20% of training	Beta distribution parameter for data mixing; scheduled off near convergence
Scheduler	Cosine Annealing	Smooth cyclical decay of learning rate over epochs (T_max = 100)
Evaluation Strategy	TTA + Confusion Matrix + F1 Score	Inference TTA: 5-crop (original + h-flip + v-flip + ±90° rotations)
Mixed-Precision Training	FP16 AMP	torch.cuda.amp.GradScaler for memory-efficient training

AdamW: Adam with decoupled Weight decay; AMP: Automatic Mixed Precision; SWA: Stochastic Weight Averaging; TTA: Test-Time Augmentation.

**Table 3:** Preprocessing and hyperparameter summary of models for used dataset.

Dataset (Source)	Preprocessing/Augmentation Techniques	Main Hyperparameters
[25]	- HU Normalization (window: −600 ~ 150)- Gaussian Blur (kernel = 5, σ = 1.0)- PreprocessingLayer (contrast = 1.5, sharpness = 1.2)- AugMix, AutoAugment, RandAugment- RandomResizedCrop, Horizontal/Vertical Flip, ColorJitter, RandomErasing (*p* = 0.3)- TTA (flip + rotation)	input size = 256, learning rate = 1 × 10^−3^, weight decay = 1 × 10^−4^, dropout rate = 0.3, base channels = 64, label smoothing = 0.1, optimizer = AdamW with OneCycleLR, maximum learning rate = 1 × 10^−2^, and early stopping patience = 50
[26]	- Gaussian Blur (kernel = 5, σ = 1.0)- PreprocessingLayer (contrast = 1.5, sharpness = 1.2)- AutoAugment, AugMix, RandAugment- RandomResizedCrop, Flip, ColorJitter, RandomErasing- TTA (flip + rotation)	input size = 256, learning rate = 1 × 10^−4^, weight decay = 1 × 10^−4^, dropout rate = 0.3, base channels = 64, label smoothing = 0.1, optimizer = AdamW with OneCycleLR, maximum learning rate = 1 × 10^−2^, and early stopping patience = 50
[27]	- AugMix, AutoAugment, RandAugment- MixUp (α = 0.4), CutMix (α = 1.0)- FGSM Adversarial Training (ε = 0.005)- RandomErasing, Flip, ColorJitter- Progressive Resizing {0:128, 50:192, 100:256}- ImbalancedDatasetSampler	number of epochs = 750, batch size = 64, learning rate = 1 × 10^−4^, weight decay = 1 × 10^−4^, dropout rate = 0.3, head dropout rate = 0.6, label smoothing = 0.1, class-balanced loss β = 0.9999, focal loss γ = 2.0, SWA learning rate = 1 × 10^−4^, and early stopping patience = 50.
[28] Imbalanced	- MixUp (α = 0.4), CutMix (α = 1.0)- Label Smoothing (0.1)- Combined Focal + CB + SCE Loss- LDAM Loss- RandAugment, AugMix, AutoAugment, RandomErasing- SAM + AdamW Optimizer- SWA + OneCycleLR	number of epochs = 750, batch size = 64, learning rate = 1 × 10^−4^, maximum learning rate = 1 × 10^−2^, weight decay = 1 × 10^−4^, dropout rate = 0.3, class-balanced loss β = 0.9999, focal loss γ = 2.0, LDAM margin = 0.5, SAM ρ = 0.05, and early stopping patience = 50
[28] Balanced	- MixUp (α = 0.4), CutMix (α = 1.0)- Label Smoothing (0.1)- Focal + CB + SCE + LDAM Loss- SAM + AdamW- SWA + OneCycleLR- AugMix, AutoAugment, RandAugment, RandomErasing (*p* = 0.3)- Progressive Resizing	number of epochs = 500, batch size = 64, learning rate = 1 × 10^−4^, maximum learning rate = 1 × 10^−2^, weight decay = 1 × 10^−4^, dropout rate = 0.3, class-balanced loss β = 0.9999, LDAM margin = 0.5, SAM ρ = 0.05, and early stopping patience = 50
Original Dataset	– HU Normalization (window: −600 ~ 150) – Random Flip, Crop, ColorJitter, GaussianBlur – RandomErasing (*p* = 0.2) – MixUp (α = 0.4, applied with *p* = 0.5) – Label Smoothing (0.1) – Class-Weighted Cross-Entropy Loss – AdamW + CosineAnnealingLR – SWA (start epoch 90) + EarlyStopping – TTA (5-crop: original + h-flip + v-flip + ±90°)	number of epochs = 100, effective batch size = 32, per-step batch size = 8, gradient accumulation steps = 4, learning rate = 1 × 10^−3^, weight decay = 1 × 10^−4^, dropout rate = 0.3, head dropout rate = 0.6, label smoothing = 0.1, class weight formula = *w_i_* = (1/*n_i_*) × *N*/*C*, gradient clipping norm = 1.0, and early stopping patience = 10

CB: Class-Balanced; SCE: Symmetric Cross-Entropy (Dataset 4 only); LDAM: Label-Distribution-Aware Margin; SAM: Sharpness-Aware Minimization; TTA: Test-Time Augmentation; SWA: Stochastic Weight Averaging; HU: Hounsfield Unit.

**Table 4:** Results of all datasets.

Dataset	Test Accuracy	COVID-19 F1	CAP F1	Lung Opacity F1	Viral Pneumonia F1	Normal F1
Maedemaftouni CT (D1)	97.45%	96.50%	99.24%	–	–	96.61%
Amanullah X-ray (D2)	93.82%	95.41%	92.89%	–	–	93.09%
Sachin X-ray (D3)	96.17%	97.87%	95.25%	–	–	95.53%
Tawsifurrahman X-ray (Imbalanced D4)	90.12%	93.63%	–	86.76%	96.23%	90.31%
Tawsifurrahman X-ray (Balanced D4)	90.60%	92.10%	–	87.10%	96.40%	87.20%
**Our Novel CT Dataset (D5)**	**99.39%**	**99.17%**	**99.13%**	**–**	**–**	**99.87%**

Bold values indicate the best performance in each metric column. The dash symbol (–) indicates that the corresponding class was not included or not applicable for that dataset.

**Table 5:** Comparison of inference latency and computational cost across backbone architectures.

Model	Params (M)	GMACs	Inference (ms)
ARTEMIS (Ours)	23.69	0.55	15.88 ± 2.35
ResNet-50	23.51	5.40	9.59 ± 1.59
DenseNet-121	6.96	3.78	24.72 ± 2.56
EfficientNet-B0	4.01	0.54	14.25 ± 3.99

**Table 6:** Detailed model complexity profile of ARTEMIS.

Metric	Value
Total parameters	23.69 M (23,688,833)
Trainable parameters	23.69 M (23,688,831)
Frozen parameters (DCAP)	2
MACs (3 × 256 × 256 input)	0.547 GMACs (547,348,992)
GFLOPs (≈2 × MACs)	1.095 GFLOPs
Model size—FP32/FP16	90.37 MB/45.18 MB

**Table 7:** Results of the ablation study.

Configuration	Accuracy	F1	McNemar p	PBS p	Perm. p	MW-U p	Wlx. p	Cohen’s d	Effect	Sig. Tests
Full ARTEMIS (baseline)	0.9847	0.9847	—	—	—	—	—	—	—	—
w/o DCAP	0.9241	0.9235	<0.001	0.0001	0.0004	0.0012	0.0009	1.18	Large	McN,PBS,Perm,MWU,Wlx
w/o Transformer	0.9592	0.9587	0.0018	0.0012	0.0024	0.0241	0.0152	0.68	Medium	McN,PBS,Perm,MWU,Wlx
w/o SE Block	0.9768	0.9762	0.0412	0.0384	0.0612	0.2840	0.1920	0.34	Small	McN,PBS

## Data Availability

The data will be available at [https://github.com/ulutashasan/YOBU_Covid_Dataset] (accessed on 10 April 2026).
